# Seasonal Hydrology Restructures Basal Carbon Pathways in a Lower Yangtze River Fish Food Web: A Stable-Isotope Baseline for the Fishing-Ban Era

**DOI:** 10.3390/biology15131076

**Published:** 2026-07-05

**Authors:** Ya Zhang, Tianshu Zhou, Yuting Zhang, Hongyi Guo, Xuguang Zhang

**Affiliations:** 1 College of Fisheries and Life Science, Shanghai Ocean University, Shanghai 201306, China; zhangya@shou.edu.cn (Y.Z.);; 2 College of Environmental and Chemical Engineering, Shanghai University of Electric Power, Shanghai 201306, China; zhoutianshu@shiep.edu.cn; 3 Engineering Technology Research Center of Marine Ranching, Shanghai Ocean University, Shanghai 201306, China

**Keywords:** stable isotope analysis, δ^13^C, δ^15^N, trophic level, isotopic niche, fish food web, Jingjiang section, Yangtze River, hydrological seasonality, ten-year fishing ban

## Abstract

Seasonal hydrology reshapes large-river food webs by altering habitat connectivity and basal resource availability. Trophic baselines from before the 2021 Yangtze ten-year fishing ban are now valuable because monitoring has shifted from fish abundance alone toward food-web function and ecological recovery. We analysed carbon (δ^13^C) and nitrogen (δ^15^N) stable isotopes of fish and the baseline bivalve *Corbicula fluminea* collected in March (dry season) and August (wet season) 2016 from the Jingjiang section of the lower Yangtze River. In the dry season, 100 individuals of 27 species were analysed; species mean δ^13^C ranged from −30.52‰ (*Micropercops swinhonis*) to −21.19‰ (*Aristichthys nobilis*) and δ^15^N from 6.30‰ (*Hypophthalmichthys molitrix*) to 14.90‰ (*Lophiogobius ocellicauda*). In the wet season, 187 individuals of 47 species were analysed; species mean δ^13^C ranged from −32.07‰ (*Pseudobrama simoni*) to −20.84‰ (*Salanx ariakensis*) and δ^15^N from 6.27‰ (*Misgurnus anguillicaudatus*) to 14.87‰ (*Saurogobio gymnocheilus*). Among 24 shared species, δ^13^C differed significantly between seasons (paired t = 4.30, *p* < 0.001), but δ^15^N did not (t = 1.52, *p* = 0.143). Mean trophic level fell from 3.07 to 2.74 (t = 3.85, *p* < 0.001). This decline remained significant in a trophic-enrichment-factor sensitivity analysis using 2.5–4.0‰. Community-wide carbon range (CR), nitrogen range (NR), total convex-hull area (TA), mean nearest-neighbour distance (NND), and the standard deviation of nearest-neighbour distance (SDNND) showed larger wet-season CR (9.08 vs. 7.51), slightly larger NR, TA and NND, and lower SDNND. Seasonal hydrology thus mainly altered basal carbon pathways and relative trophic positions rather than reorganising feeding guilds. The dataset provides a pre-ban isotopic baseline for assessing whether post-ban recovery in the lower Yangtze includes restoration of trophic structure and energy-flow pathways.

## Simple Summary

Large rivers change greatly between dry and wet seasons, and fish may respond by shifting their food sources. Understanding these seasonal changes is important for judging whether the Yangtze River is recovering under the current ten-year fishing ban. This study used carbon and nitrogen stable isotopes to describe the fish food web in the Jingjiang section of the lower Yangtze River before the ban began. Fish and a clam baseline were sampled during the dry (March) and wet (August) seasons of 2016. Carbon isotope values shifted strongly between seasons, showing that fish relied more on carbon sources depleted in ^13^C during the wet season, when floods increase habitat connectivity and bring in outside organic matter. Nitrogen isotope values changed less, indicating that broad feeding roles remained relatively stable. Additional robustness checks showed that the seasonal trophic level decline was insensitive to trophic enrichment factors of 2.5–4.0‰. These results provide a pre-ban reference for future monitoring and show that post-ban assessments should consider not only fish abundance and diversity, but also whether energy pathways and trophic interactions are being restored.

## 1. Introduction

The Yangtze River ranks among the largest and most heavily exploited river systems globally. Its fish assemblages have declined sharply over recent decades under the combined pressure of overfishing, dam construction, channel modification, pollution, and habitat loss. In 2021, the Chinese government imposed a basin-wide ten-year commercial fishing ban—an unprecedented measure for a river of this scale. Early monitoring data from the mainstem and tributaries indicate initial gains in fish biomass, body condition, and species diversity, though the pace of recovery varies among reaches and taxonomic groups [1,2,3,4,5,6].

The lower Yangtze, where riverine and estuarine habitats overlap, is a critical zone for tracking these changes. The Jingjiang section sits near the river mouth and is shaped by tidal currents, high sediment loads, and large seasonal differences in discharge. Historically, the reach has supported an assemblage of migratory, estuarine-associated, and resident freshwater fishes, including commercially important species (*Coilia nasus*, *Siniperca chuatsi*, *Pelteobagrus fulvidraco*, *Channa argus*, *Hypophthalmichthys molitrix*, and *Aristichthys nobilis*) and conservation- or management-relevant taxa such as *Coilia nasus*, *Anguilla japonica*, *Takifugu obscurus*, and several Siniperca species [7]. The flooding season, roughly May to October, brings elevated water levels, greater lateral connectivity with floodplains, and increased terrestrial input. The dry season, from November to April, reduces these inputs and contracts available habitat. Such hydrological variation can shift the basal resources that underpin the fish food web.

Stable isotope analysis offers a time-integrated view of trophic relationships that complements instantaneous dietary methods such as stomach contents. Carbon isotope ratios (δ^13^C) change relatively little between prey and consumer and thus track the origin of assimilated carbon, while nitrogen isotope ratios (δ^15^N) increase by roughly 2–5‰ per trophic step and are used to estimate trophic position [8,9,10,11]. In dynamic systems where primary producers turn over rapidly, long-lived primary consumers such as bivalves provide a more stable isotopic baseline for trophic calculations [9,12].

At the community level, species positions in δ^13^C-δ^15^N space can be described by a set of quantitative metrics [13,14]: carbon range (CR) and nitrogen range (NR) capture the breadth of basal resources and vertical food-chain length; convex-hull area (TA) and mean distance to centroid (CD) measure overall trophic diversity; and nearest-neighbour distance (NND) and its standard deviation (SDNND) indicate the degree of trophic redundancy and evenness. Bayesian ellipse methods and mixing models have since extended this toolkit [15,16,17,18], but the original Layman metrics remain widely used for comparative purposes. These approaches have been applied in estuarine, coastal, and freshwater food webs worldwide [19,20,21,22,23,24,25,26,27,28,29,30,31,32], including Chinese waters and the Yangtze estuary [33,34,35,36]. Compared with Yangtze estuary studies, the Jingjiang section represents an upstream, predominantly freshwater but tidally influenced transition reach; salinity effects are weaker, resident freshwater fishes are more common, and seasonal discharge and lateral connectivity are expected to exert stronger control over basal resource routing than marine–estuarine mixing.

Hydrological seasonality is a major structuring force in floodplain and large-river food webs. Work in Poyang Lake, Dongting Lake, Lake Taihu, the Chishui River, and other Yangtze tributaries shows that water-level change can shift the balance between pelagic, littoral, and terrestrial carbon pathways reaching fish [37,38,39,40,41,42,43,44,45,46,47,48,49,50]. The magnitude and direction of these shifts, however, depend on local geomorphology, connectivity, and assemblage composition, so generalisations from one system to another are not straightforward.

Here, we use δ^13^C and δ^15^N to compare fish food-web structure between dry and wet seasons in the Jingjiang section. We examine seasonal changes in isotope values, trophic levels across feeding guilds, and community-wide niche metrics for the 24 species shared between seasons. Our samples were collected in 2016—five years before the fishing ban—and therefore serve as a pre-ban trophic baseline. We expected the wet season to broaden the range of carbon sources used by fish because of increased habitat connectivity and allochthonous input, while anticipating that trophic level shifts would be moderate given that most species maintain similar feeding habits year-round.

## 2. Materials and Methods

### 2.1. Study Area and Sample Collection

**Figure 1:**
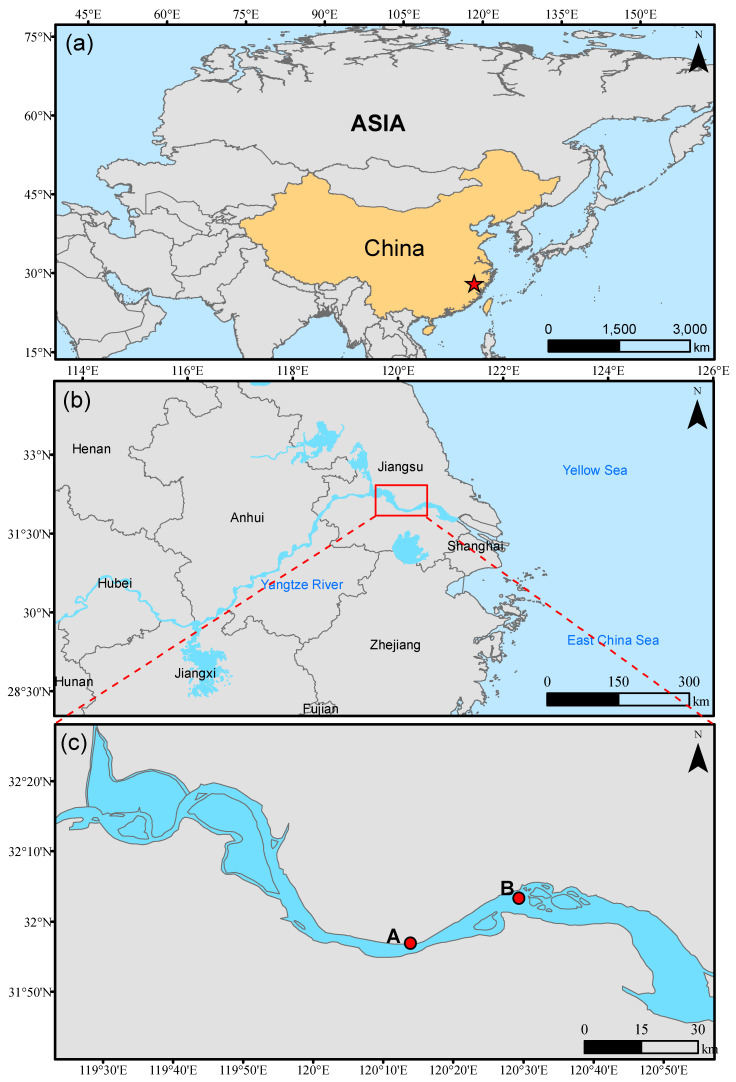
Location of the Jingjiang section and fixed sampling sites in the lower Yangtze River, China. (**a**) The study area in the broader Asian context, with China highlighted in yellow and the approximate location of Jingjiang marked by a red star. (**b**) Regional position of the Jingjiang section within the lower Yangtze River and adjacent estuarine region; the red rectangle indicates the enlarged area shown in panel (**c**), and the red dashed lines link the regional and enlarged maps. (**c**) Enlarged map of the Jingjiang reach showing the two fixed sampling areas used during the March 2016 dry-season and August wet-season sampling campaigns. Sites A and B indicate the near-bank and Jiangxin Island sampling areas, respectively. Blue areas represent water bodies.

The sampling station was located on the north bank of the lower Yangtze River near Jingjiang City, Jiangsu Province, China (31°56′ N, 120°01′ E; Figure 1), about 240 km upstream of the river mouth. Water level at the site is influenced by both upstream Yangtze discharge and estuarine tidal forcing. The local tide is irregular semidiurnal, but salinity remained effectively zero throughout the study. Mid-channel sandbars immediately upstream and downstream create locally complex flow and a sheltered nearshore habitat. The shoreline grades from a concrete dyke at the upper margin, through an approximately 30 m riprap band at the dyke toe, to an approximately 100 m belt of *Phragmites australis* between the riprap and the mudflat edge. The reeds reach about 3 m in summer and autumn and are cleared in winter. The reach lies within the National Aquatic Germplasm Resource Conservation Area for Chinese mitten crab (*Eriocheir sinensis*) and mandarinfish (*Siniperca chuatsi*). In Figure 1, Site A denotes the north-bank nearshore station and Site B denotes the Jiangxin Island station; the two sampling areas are approximately 38 km apart along the lower Yangtze reach.

Fish were collected in March and August 2016 using T-shaped fixed trap nets deployed at Site A along the bank and Site B near Jiangxin Island in the Jingjiang section (Figure 1). One T-shaped fixed trap net was deployed at each sampling area. March samples represented the dry season, and August samples the wet season. Each T-shaped fixed trap net consisted of a 40 m lead panel set perpendicular to the riverbank and two cage-like cod-end bag nets fixed near the riverward lower edge of the lead panel. The lead panel had a mesh size of 1.9 cm, a vertical height of approximately 1.8 m, a lower rope buried in the mudflat substrate, and an upper rope kept near the mean water surface by seasonal adjustment with water level. The two cod-end bag nets were approximately 10 m long each and contained internal guiding panels that allowed fish to move one way into the cod ends; the cod-end mesh size was 0.9 cm. The passive nets were left in place continuously (24 h per day) and cleared once daily after the afternoon ebb tide, so each retained catch represented approximately one daily soak interval. Damaged or fouled gear was replaced with nets of identical specification. The same gear design, mesh sizes, and clearing protocol were used in both seasons to improve comparability between dry- and wet-season samples. Sampling effort was explicitly standardised between seasons: in both the March dry-season and August wet-season campaigns, the same two T-shaped trap nets were fished continuously for 24 h per day at the two sampling areas, cleared once daily after ebb tide, and operated for approximately two weeks (about 14 sampling days). Thus, the cumulative soak effort was approximately 672 net-hours per season (2 nets × 14 days × 24 h), equivalent to about 28 net-days per season, so the dry- and wet-season catches were obtained under essentially equivalent gear-deployment effort. *Corbicula fluminea* collected alongside fish was used as the trophic baseline, because long-lived primary consumers integrate local baseline isotope variation more effectively than short-lived resources such as seston or detritus [9,12]. Because *C. fluminea* primarily integrates benthic and suspended particulate pathways, the single-baseline design may not fully represent pelagic, littoral, or floodplain baselines used by mobile fishes. The study therefore represents a pre-ban isotopic baseline for this lower Yangtze reach.

### 2.2. Sample Processing and Stable-Isotope Analysis

In the laboratory, fish total length and body mass were measured, and species were identified using regional taxonomic monographs [51]. Dorsal white muscle was dissected from each fish, dried to constant mass, ground into a homogeneous powder and stored dry. Mature *Corbicula fluminea* individuals used as baseline organisms had shell lengths of 28–38 mm, shell heights of 27–35 mm, and shell widths of 20–29 mm. They were held overnight in distilled water for gut clearance, after which soft tissues were dissected. Powdered samples were acidified with 1 M hydrochloric acid to remove inorganic carbonates until CO_2_ release ceased, rinsed with distilled water, oven-dried at 60 °C for 48 h, and ground with an agate mortar and pestle. Fish muscle C:N mass ratios were screened to evaluate whether lipid correction was required for δ^13^C interpretation.

Stable isotope analyses were conducted at the Instrumental Analysis Centre of Shanghai Jiao Tong University using an elemental analyser coupled to an isotope-ratio mass spectrometer (Vario EL III/Isoprime, Elementar Analysensysteme GmbH, Hanau, Germany). Two laboratory standards were measured after every 48 samples to monitor accuracy and instrumental drift. Stable isotope values are reported in delta notation (‰) relative to international standards: δX = [(R_sample/R_standard) − 1] × 1000, where X is ^13^C or ^15^N and R is the corresponding ^13^C/^12^C or ^15^N/^14^N ratio. Samples were analysed in duplicate; analytical precision was better than ±0.1‰ for δ^13^C and ±0.2‰ for δ^15^N.

### 2.3. Trophic-Level and Community-Wide Isotope Metrics

Trophic levels (TLs) were estimated from fish δ^15^N values relative to the season-specific C. fluminea baseline using TL = [(δ^15^N_consumer − δ^15^N_baseline)/TEF] + λ, where λ is the trophic level of the baseline organism (set to 2) and TEF is the trophic enrichment factor. We used a TEF of 3.4‰ as the primary value, because it is widely adopted in aquatic food-web studies [9,10]. To address uncertainty in trophic enrichment, we also performed a sensitivity analysis using TEF values of 2.5 and 4.0‰. The C. fluminea baseline δ^15^N values used in the calculations were 7.42‰ in the dry season (*n* = 8) and 8.10‰ in the wet season (*n* = 10).

Community-wide trophic niche structure was summarised using six Layman metrics calculated from unweighted species’ mean positions in δ^13^C-δ^15^N space for the 24 species shared by both seasons [13,14]: δ^15^N range (NR), δ^13^C range (CR), total convex-hull area (TA), mean distance to centroid (CD), mean nearest-neighbour distance (NND), and the standard deviation of nearest-neighbour distance (SDNND). NR reflects vertical trophic structure, CR reflects basal carbon-source diversity, TA and CD reflect overall trophic diversity and dispersion, and NND and SDNND describe trophic redundancy and evenness among species.

### 2.4. Statistical Analysis

Species-level mean isotope values were used for seasonal comparisons. Dry- and wet-season δ^13^C, δ^15^N, and trophic-level values were compared for the 24 species present in both seasons using paired t-tests after checking the normality of within-species differences with Shapiro–Wilk tests. Species represented by a single individual in one season were retained in paired analyses because the statistical unit was the species mean; their low within-season replication is acknowledged as a limitation. Guild-level differences in δ^13^C, δ^15^N, and trophic level were tested within each season using Kruskal–Wallis tests, followed by Holm-adjusted pairwise Mann–Whitney tests when appropriate. Community-wide metrics were calculated from the isotope biplot coordinates of shared species. Analyses were performed using Python 3.11.9 (NumPy 1.26.4, SciPy 1.13.1, and pandas 2.2.2) and checked against the original R 4.4.1 computations using the SIAR package.

Broad feeding guilds (herbivorous, omnivorous and carnivorous) were assigned from regional taxonomic monographs and published diet studies [51]. Two guild assignments were updated relative to earlier analyses: *Acheilognathus macropterus* was reclassified as omnivorous and *Megalobrama skolkovii* as herbivorous, following re-examination of diet information in the original monographs.

## 3. Results

### 3.1. Seasonal Variation in Fish δ^13^C and δ^15^N

Carbon and nitrogen stable-isotope values for fish collected in the dry and wet seasons are provided in Table 1. In the dry season, 100 fish individuals representing 27 species were analysed. Species mean δ^13^C values ranged from −30.52‰ in *Micropercops swinhonis* to −21.19‰ in *Aristichthys nobilis*, with an assemblage mean of −24.82‰. Species mean δ^15^N values ranged from 6.30‰ in *Hypophthalmichthys molitrix* to 14.90‰ in *Lophiogobius ocellicauda*, with an assemblage mean of 11.26‰.

In the wet season, 187 fish individuals representing 47 species were analysed. Species mean δ^13^C values ranged from −32.07‰ in *Pseudobrama simoni* to −20.84‰ in *Salanx ariakensis*, with an assemblage mean of −26.12‰. Species mean δ^15^N values ranged from 6.27‰ in *Misgurnus anguillicaudatus* to 14.87‰ in *Saurogobio gymnocheilus*, with an assemblage mean of 10.98‰. The wet-season assemblage included more species and showed a broader spread of carbon isotope values.

**Figure 2:**
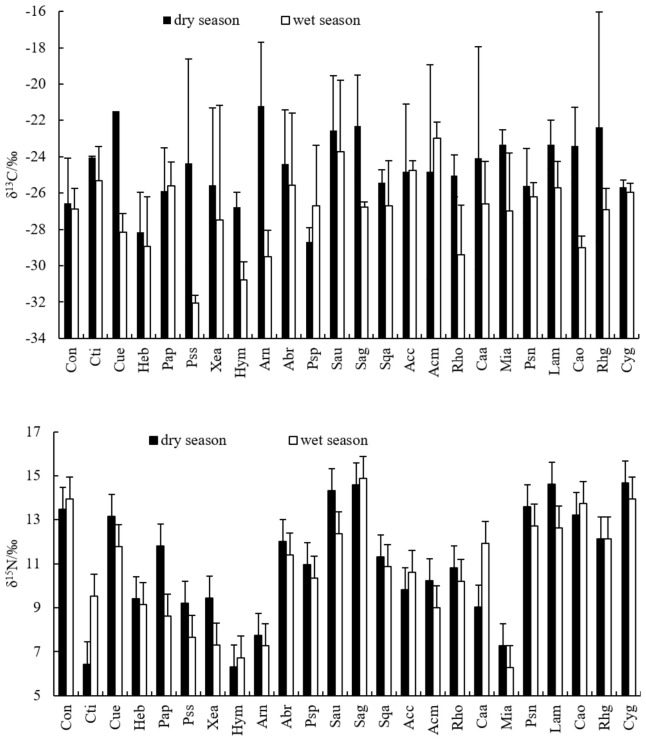
Seasonal variation in species mean δ^13^C and δ^15^N values for the 24 fish species common to the March dry-season and August wet-season sampling campaigns in the Jingjiang section of the lower Yangtze River, China, in 2016. Bars represent species means, vertical error bars indicate standard deviations, and species abbreviations follow the species ID codes listed in Table 1.

Twenty-four species were common to both seasons (Figure 2). Shapiro–Wilk tests indicated that within-species seasonal differences did not deviate significantly from normality for δ^13^C (*p* = 0.326), δ^15^N (*p* = 0.120) or trophic level (*p* = 0.116). Paired comparisons showed a significant seasonal shift in δ^13^C (paired t-test, t = 4.30, *p* < 0.001), whereas δ^15^N did not differ significantly (t = 1.52, *p* = 0.143). Mean δ^13^C of shared species shifted from −24.58‰ in the dry season to −27.03‰ in the wet season, indicating greater reliance on ^13^C-depleted carbon pathways during high water. Mean δ^15^N changed more modestly, from 11.07‰ to 10.62‰, confirming that seasonal changes in basal carbon sources were more pronounced than changes in raw nitrogen isotope position.

### 3.2. Isotope Structure by Feeding Guild

**Figure 3:**
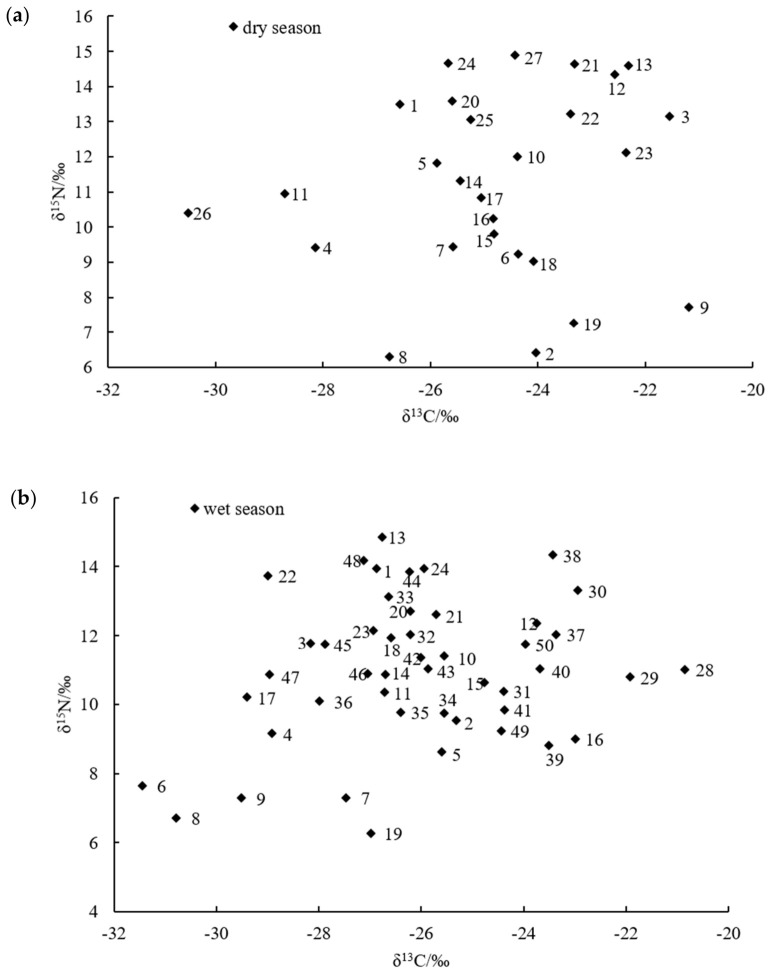
Species mean δ^13^C–δ^15^N isotope space for fish collected from the Jingjiang section of the lower Yangtze River, China, in 2016: (**a**) March dry season and (**b**) August wet season. Each point represents the mean isotope position of one species, and numbers indicate the species ID codes listed in Table 1.

**Figure 4:**
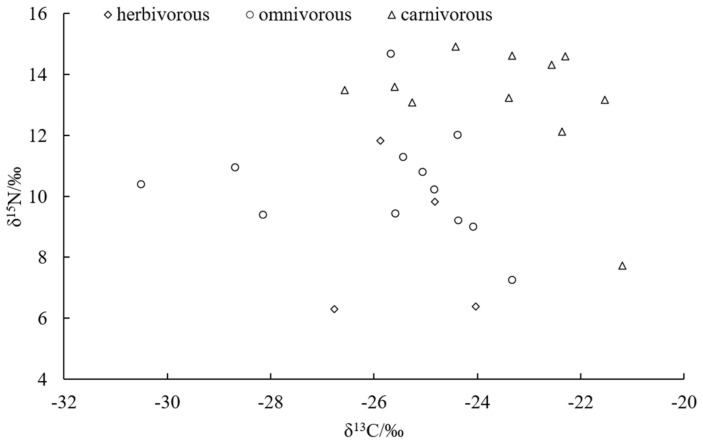
δ^13^C–δ^15^N isotope space of fish species grouped by feeding guild in the Jingjiang section of the lower Yangtze River, China, based on samples collected during the March dry-season and August wet-season campaigns in 2016. Diamonds indicate herbivorous species, circles indicate omnivorous species, and triangles indicate carnivorous species; feeding-guild assignments follow the sources cited in Table 1.

During the dry season, the assemblage comprised four herbivorous, twelve omnivorous, and eleven carnivorous species (Figure 3 and Figure 4). Mean δ^13^C values for herbivorous, omnivorous, and carnivorous fishes were −25.37‰, −25.84‰, and −23.50‰, respectively, and mean δ^15^N values were 8.59‰, 10.40‰, and 13.16‰, respectively. Kruskal–Wallis tests showed significant among-guild differences in dry-season δ^13^C (H = 6.64, *p* = 0.036), δ^15^N (H = 9.53, *p* = 0.009) and trophic level (H = 9.53, *p* = 0.009). Carnivorous fishes therefore occupied significantly higher nitrogen-isotope and trophic-level space, whereas carbon separation among herbivorous and omnivorous fishes was limited.

During the wet season, five herbivorous, seventeen omnivorous, and twenty-five carnivorous species were recorded. Mean δ^13^C values for herbivorous, omnivorous, and carnivorous fishes were −26.62‰, −26.50‰, and −25.75‰, respectively, and mean δ^15^N values were 9.72‰, 9.93‰, and 11.94‰, respectively. Wet-season guilds did not differ significantly in δ^13^C (Kruskal–Wallis H = 1.33, *p* = 0.513), but they differed significantly in δ^15^N (H = 11.92, *p* = 0.003) and trophic level (H = 11.92, *p* = 0.003). Thus, guild identity remained most evident along the nitrogen and trophic-level axes, whereas carbon sources overlapped more strongly among guilds during high-water conditions.

Post hoc pairwise comparisons supported the same pattern. In the dry season, carnivores differed from herbivores in δ^15^N and trophic level after Holm correction (*p* = 0.024), whereas guild differences in δ^13^C were weaker. In the wet season, carnivores differed from herbivores (*p* = 0.006) and omnivores (*p* = 0.006) for δ^15^N and trophic level. These tests confirm that the higher trophic position of carnivores was statistically supported rather than only visually inferred from the isotope plots.

### 3.3. Seasonal Dynamics of Fish Trophic Levels

**Figure 5:**
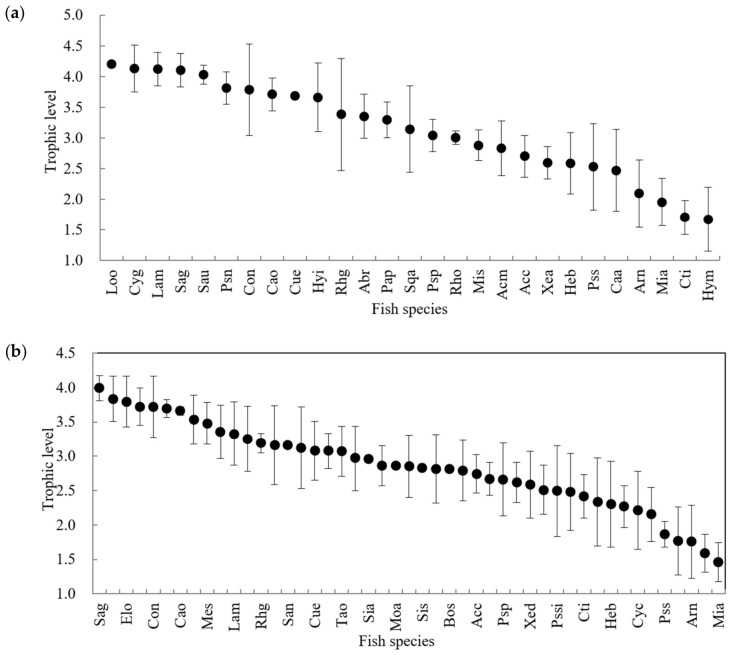
Estimated trophic levels of fish species collected from the Jingjiang section of the lower Yangtze River, China, during the 2016 March dry-season and August wet-season sampling campaigns: (**a**) March and (**b**) August. Trophic levels were calculated using season-specific *Corbicula fluminea* as the primary-consumer baseline and a δ^15^N trophic enrichment factor of 3.4‰. Species are ordered from high to low trophic level within each season, and vertical error bars indicate standard deviations.

Estimated trophic levels of the 27 dry-season species ranged from 1.67 in H. molitrix to 4.20 in *L. ocellicauda*, with a mean of 3.13 (Figure 5). Five species exceeded a trophic level of 4.0: *L. ocellicauda*, *Cynoglossus gracilis*, *Lateolabrax maculatus*, *S. gymnocheilus*, and *Saurogobio dabryi*. Eleven species fell between 3.0 and 4.0, eight species between 2.0, and 3.0, and three species below 2.0. Values below 2.0 should not be interpreted as true positions below primary consumers; they indicate uncertainty associated with baseline choice, TEF assumptions, and spatially heterogeneous δ^15^N baselines. Mean trophic levels of herbivorous, omnivorous, and carnivorous fishes were 2.34, 2.88, and 3.69, respectively.

In the wet season, trophic levels of the 47 species ranged from 1.46 in *M. anguillicaudatus* to 3.99 in *S. gymnocheilus*, with a mean of 2.85 (Figure 5). Nineteen species fell between 3.0 and 4.0, twenty-three between 2.0 and 3.0, and five below 2.0. Mean trophic levels of herbivorous, omnivorous, and carnivorous fishes were 2.48, 2.54, and 3.13, respectively. The wet-season assemblage therefore contained more species but had a lower overall mean trophic level, although the low-TL estimates again require cautious interpretation because of single-baseline uncertainty.

**Figure 6:**
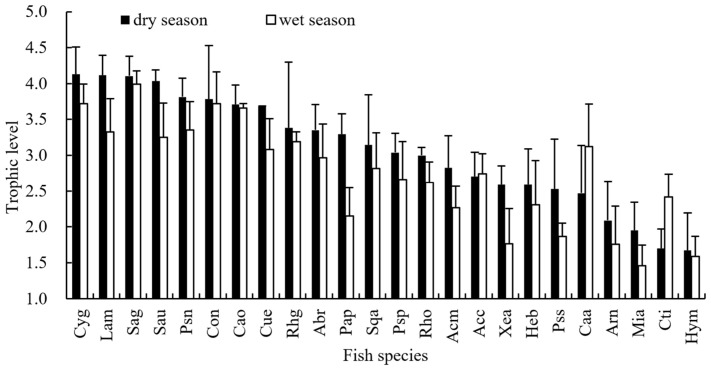
Seasonal trophic-level changes in the 24 fish species collected in both the March dry-season and August wet-season sampling campaigns in the Jingjiang section of the lower Yangtze River, China, in 2016. Trophic levels were estimated using season-specific *Corbicula fluminea* as the primary-consumer baseline and a δ^15^N trophic enrichment factor of 3.4‰; vertical error bars indicate standard deviations. Species abbreviations follow the species ID codes listed in Table 1.

For the 24 species collected in both seasons, mean trophic level declined significantly from 3.07 in the dry season to 2.74 in the wet season (paired *t*-test, t = 3.85, *p* < 0.001), an average decrease of 0.33 trophic-level units (Figure 6). The TEF sensitivity analysis confirmed that this conclusion was robust: the mean dry-minus-wet decline was 0.45 trophic level units with TEF = 2.5‰, 0.33 with TEF = 3.4‰, and 0.28 with TEF = 4.0‰, with significant paired differences in all cases (*p* < 0.001). *Carassius auratus* and *Ctenopharyngodon idella* increased in trophic level during the wet season, *Acheilognathus chankaensis* showed negligible change (+0.04), and most other shared species decreased to varying degrees. The largest decline occurred in *Parabramis pekinensis*, which decreased by approximately one trophic level unit. Across shared species, trophic levels remained ordered as herbivorous < omnivorous < carnivorous in both seasons, indicating that seasonal hydrology shifted relative trophic positions without erasing broad feeding-guild structure.

Across the 24 shared species, the TEF sensitivity analysis also retained the guild ordering under all enrichment values tested. With TEF = 2.5‰, mean dry-season and wet-season trophic levels were 3.46 and 3.01; with TEF = 4.0‰, they were 2.91 and 2.63. Thus, changing TEF altered the absolute trophic level estimates but not the direction or significance of the seasonal pattern. Across the full assemblage, the same TEF sensitivity range bracketed individual species trophic levels between 1.27 and 4.99 at TEF = 2.5‰ and between 1.54 and 3.87 at TEF = 4.0‰ (1.46–4.20 at the primary TEF of 3.4‰). The overall envelope of estimated trophic level variation under TEF = 2.5–4.0‰; therefore, it spanned approximately 1.3 to 5.0 trophic level units.

### 3.4. Community-Wide Isotope Metrics

Community-wide isotope metrics calculated for the 24 species common to both seasons are shown in Table 2. NR was slightly higher in the wet season (8.60‰) than in the dry season (8.37‰), suggesting a modest expansion of vertical trophic structure. CR was substantially higher in the wet season (9.08‰ vs. 7.51‰), indicating broader basal carbon-source diversity during high-water conditions. TA was slightly higher in the wet season (49.57 vs. 48.28), and CD changed only marginally (2.88 vs. 2.86). NND increased from 1.02 to 1.11, and SDNND decreased from 0.61 to 0.53 in the wet season. Together, these metrics indicate that wet-season hydrology broadened the carbon and nitrogen ranges and slightly expanded overall niche area, while nearest-neighbour distances were distributed more evenly.

## 4. Discussion

### 4.1. Seasonal Hydrology and Basal Carbon Pathways

The clearest seasonal pattern was a shift toward lower δ^13^C in the wet season, evident both across the full assemblage and in the 24 shared species. Since δ^13^C fractionation during trophic transfer is small [8,9], this decline points to a change in the carbon sources assimilated by fish rather than a change in trophic level itself. During high discharge, lateral exchange between the channel and its margins intensifies, terrestrial detritus and floodplain organic matter enter the aquatic food web in greater quantities, and suspended particulate matter becomes more ^13^C-depleted. These processes account for the lower wet-season δ^13^C and the wider CR we observed.

Similar carbon-source shifts have been reported from other Yangtze-connected water bodies. In Poyang Lake, Wang et al. [37,38] found that water-level change altered the relative contribution of seston, macrophytes, and terrestrial C_3_ plant material to consumers. Zhang et al. [39] reported wider δ^13^C ranges during the wet season in Dongting Lake, and Zhou et al. [40] and Xu et al. [41] documented seasonal isotope shifts in Lake Taihu. Studies in Yangtze tributaries and oxbows confirm that high water tends to expand the suite of carbon sources available to fish [44,45,47]. The Jingjiang data fit this regional picture.

That δ^13^C responded more strongly than δ^15^N suggests that hydrological seasonality has a larger effect on the routing of basal carbon than on vertical food-chain position. Some species showed particularly large δ^13^C shifts: *Pseudobrama simoni* (−7.70‰), *Aristichthys nobilis* (−8.32‰), and *Hypophthalmichthys molitrix* (−4.01‰), all of which probably increased their intake of planktonic or detrital resources during the flood period. However, two-isotope data alone cannot resolve the specific contributions of individual sources. Although the raw dataset includes several candidate basal materials, the available source isotope data are not sufficiently comprehensive or well separated to support a robust Bayesian mixing model. Future work should sample seston, periphyton, macrophytes, riparian C_3_ plants, floodplain detritus, and benthic invertebrates directly in each season, and apply Bayesian mixing models only when source discrimination and replication are adequate [16,17].

### 4.2. Nitrogen Isotope Patterns, Trophic Levels and Baseline Choice

Although raw δ^15^N did not differ significantly between seasons, baseline-corrected trophic levels declined in the wet season. This seeming contradiction arises because consumer δ^15^N reflects both diet and the isotopic baseline, so if the baseline itself shifts seasonally, raw values can mask real changes in trophic position [9,18,42]. We used *Corbicula fluminea* as the baseline, which is standard practice [12], but this species integrates primarily the benthic and suspended-particulate pathways and may not fully represent the pelagic, littoral, or floodplain baselines that mobile fishes exploit. This limitation is especially relevant in a tidally influenced, seasonally connected large-river reach where fishes can move among channel, margin, and floodplain habitats.

The moderate trophic level decline—on average 0.33 units, with most species changing by less than 0.5—probably reflects several reinforcing processes. In the dry season, reduced habitat volume concentrates prey and predators, and benthic or piscivorous feeding may be favoured. In the wet season, an expanded habitat allows fishes to disperse and to feed on more abundant lower-trophic resources such as detritus, periphyton, and small invertebrates. Ontogenetic diet shifts may also contribute in individual species if wet-season catches include smaller carnivores. In our body-size check, however, shared carnivorous species as a group were not significantly smaller in the wet season (mean total length 12.32 cm) than in the dry season (12.33 cm; Mann–Whitney *p* = 0.811), although some species such as *Lateolabrax maculatus* and *Saurogobio dabryi* were smaller in the wet season. Thus, ontogeny may contribute to species-specific shifts but is unlikely to fully explain the community-wide decline. Only *Parabramis pekinensis* dropped by about one full trophic level, likely because this herbivore shifts to markedly different plant resources between seasons. The broad ordering—herbivores below omnivores below carnivores—held across both seasons, indicating plasticity in trophic position within, not between, feeding guilds.

A few estimated trophic levels fell below 2.0 (e.g., H. molitrix at 1.67 in the dry season, M. anguillicaudatus at 1.46 in the wet season). These values are not biologically meaningful as positions below primary consumers; they reflect baseline mismatch, spatial heterogeneity in δ^15^N, or the inherent uncertainty of using a single TEF value. The TEF sensitivity analysis showed that low-TL estimates persisted under TEF values of 2.5–4.0‰, confirming that the issue is not caused solely by the selected 3.4‰ enrichment factor. A TEF of 3.4‰ is a useful default [10] but can deviate considerably depending on diet quality and consumer physiology [11]. Fish muscle C:N mass ratios were low (maximum approximately 3.68), so a lipid correction for δ^13^C was not applied; nevertheless, isotopic routing and tissue-specific turnover may add uncertainty to the interpretation of species-specific values. Collecting multiple baseline taxa (e.g., snails, shrimp, zooplankton) in each season and running sensitivity analyses with a range of TEF values would strengthen future estimates.

### 4.3. Community-Wide Trophic Structure and Feeding-Guild Stability

The community-wide metrics tell a coherent story. The larger wet-season CR (9.08 vs. 7.51) confirms broader basal resource diversity under high water. NR increased only slightly (8.60 vs. 8.37), suggesting that vertical food-chain length did not change much. TA and CD rose marginally, and NND increased while SDNND decreased—meaning that species were spaced more evenly in isotope space during the wet season rather than clustering around a few resource pathways.

In the dry season, higher SDNND may reflect uneven packing of species around limited food sources. When water levels rise and new habitats become accessible, some species can spread into previously unavailable niches, evening out the spacing. Abrantes et al. [22] and Ru et al. [48] reported analogous metric-level shifts under seasonal or regulated hydrological change, confirming that water-level variation can reshuffle trophic diversity without necessarily altering every dimension of niche structure. Taken together, the Jingjiang food web appears seasonally flexible but structurally conservative.

The guild-level data support the same conclusion. Carnivores consistently occupied higher δ^15^N positions than omnivores and herbivores in both seasons; what changed was δ^13^C, which varied more within and among guilds. This pattern is expected when guild identity constrains trophic height while hydrology controls basal carbon routing. Some carnivorous species nevertheless plotted close to or below omnivorous or herbivorous species in δ^15^N or trophic level, a pattern that likely reflects species-specific diet breadth, ontogeny, spatial baseline variation, and the fact that broad literature-based guilds do not capture short-term assimilated resources. Species richness nearly doubled in the wet season (47 vs. 27 species). Wet-season additions included estuarine-associated or migratory taxa such as *Salanx ariakensis*, *Salanx cuvieri*, *Anguilla japonica*, and *Takifugu obscurus*, as well as additional resident benthic and piscivorous species. Because sampling effort was standardised between seasons (the same two trap nets, continuous 24 h deployment, once-daily clearing, and approximately 14 sampling days per season; Section 2.1), unequal netting effort is unlikely to explain this increase. The near-doubling of species richness is therefore more plausibly interpreted as seasonal habitat expansion and the movement of estuarine-associated and migratory taxa into the reach during high water. Nevertheless, because each season was represented by a single approximately two-week campaign in one year, and because passive-gear catchability can still vary with hydrology and fish movement, the richness contrast should be interpreted with caution. The paired analysis of 24 shared species therefore provides the cleanest test of seasonal effects, independent of compositional turnover.

### 4.4. Significance of the 2016 Baseline in the Yangtze Fishing-Ban Era

When these samples were collected in 2016, the study was a regional food-web description. The 2021 fishing ban has changed the context: the same data now constitute a functional pre-ban baseline. Xiong et al. [1] recently demonstrated that the ban has reversed seven decades of fish biomass decline across much of the Yangtze mainstem, and additional studies have documented early recovery signals in diversity, body condition, and community structure [2,3,4,5,6]. Yet abundance and diversity alone do not reveal whether energy pathways and predator–prey linkages are being restored.

Isotope baselines can fill this gap. If post-ban sampling in the Jingjiang section finds more large-bodied or high-trophic fishes, isotope data can distinguish genuine food-web expansion from a simple reshuffling of the same species list. Changes in δ^13^C range would show whether newly connected habitats are contributing additional energy channels. Our 2016 data define the seasonal envelope of pre-ban trophic variation: wet-season fishes were ^13^C-depleted, trophic levels dropped modestly, and niche metrics shifted without fundamentally reorganising the food web. This seasonal envelope provides a direct benchmark for testing whether post-ban changes exceed natural dry–wet variation.

For the ban to be judged ecologically successful, recovery should extend beyond numbers of fish to include multiple basal resource pathways, adequate trophic redundancy, and functioning predator–prey interactions. The Jingjiang section, situated at the estuarine transition, may follow a different recovery trajectory from upstream tributaries or connected lakes. Repeating this isotope survey post-ban, using the same seasons, comparable gear, and additional baseline organisms, would allow the principal signatures of functional recovery against the 2016 baseline—an expanded predator δ^15^N space, maintained or broadened basal-carbon diversity, and increased trophic redundancy—to be distinguished from a purely numerical increase in abundance or richness. The specific quantitative thresholds implied by each of these signatures are set out below. That comparison would help separate the effects of the fishing ban from those of hydrological management, habitat restoration, and water quality change [3,51].

These hypotheses convert the pre-ban baseline into a testable monitoring framework. As a concrete, quantitative example, the 2016 baseline yields directly testable thresholds for post-ban monitoring conducted in comparable seasons with equivalent gear. First, for energy pathway coupling, one testable expectation is that post-ban wet-season carbon range (CR) should remain comparable to or greater than the 2016 wet-season value of 9.08‰, rather than contracting toward or below the dry-season value of 7.51‰; a contraction toward or below 7.51‰ would instead be consistent with simplification of basal resource pathways. Second, for predator–prey structure, the return of large predators should increase the frequency, biomass, or isotopic representation of high-trophic species and may raise the maximum community trophic level above the 2016 upper bound of 4.20 (at TEF = 3.4‰) and the nitrogen range (NR) beyond the 2016 envelope of 8.37–8.60‰. Third, for trophic redundancy, genuine functional recovery predicts a decrease in mean nearest-neighbour distance (NND) below the 2016 range of 1.02–1.11 without a concurrent rise in SDNND above approximately 0.6, the latter of which would signal niche compression rather than redundancy. By contrast, a purely numerical recovery predicts that abundance and species richness increase while CR, NR, NND, and the trophic-level envelope remain statistically indistinguishable from these 2016 values. Incorporating Bayesian standard ellipse areas (SEAc) in future surveys would add a continuous, statistically comparable index of isotopic niche change to test against this baseline. A post-ban food web would be considered functionally recovered only if changes in abundance and species richness are accompanied by isotopic evidence for restored predator–prey structure, sustained use of multiple basal resource pathways, and trophic redundancy that remains within or improves upon the 2016 seasonal range.

### 4.5. Limitations and Future Work

Several limitations should be kept in mind. Sampling consisted of a single approximately two-week campaign per season within one year. Although gear type, deployment duration, and net-hours were standardised and closely comparable between seasons (Section 2.1), this design cannot separate seasonal effects from year-to-year variability, and passive-gear catchability may still differ between low- and high-water conditions. Some species had small sample sizes (*n* = 1 or 2), which weakens guild-level averages and species-specific inference, although the paired tests were conducted at the species-mean level. We relied on a single baseline organism and a primary TEF of 3.4‰, both of which introduce uncertainty, even though the TEF sensitivity analysis supported the main seasonal conclusion. Although the sampled *C. fluminea* individuals were within a restricted shell-size range, the single-baseline design cannot fully capture spatial or pathway-specific baseline variation in a heterogeneous large river. Two-isotope data also cannot fully separate overlapping basal sources.

Future studies should incorporate multi-year seasonal sampling, the same fully documented net-hour standardisation used here, additional baseline taxa, direct measurements of basal resources, bivalve shell-size records, body-size data, stomach contents or DNA metabarcoding, and Bayesian mixing models when source data are sufficiently constrained. Coupling these approaches with post-ban fishery monitoring would clarify whether biomass recovery is accompanied by longer food chains, greater trophic redundancy, or shifts in basal resource dependence. Despite its limitations, this dataset offers a rare pre-ban isotopic snapshot of the lower Yangtze fish community and a practical reference point for evaluating functional recovery.

## 5. Conclusions

Seasonal hydrology shaped the Jingjiang fish food web primarily by altering basal carbon pathways. Wet-season fishes were more ^13^C-depleted, and the 24 shared species showed broader carbon and nitrogen isotope ranges, slightly larger total niche area, and more even trophic spacing, while mean trophic level declined by 0.33 units. This trophic level decline remained significant when TEF values of 2.5–4.0‰ were used. The broad ordering of herbivorous < omnivorous < carnivorous fishes held across both seasons, although TL values below 2.0 highlight baseline uncertainty in a spatially heterogeneous river. Because sampling preceded the Yangtze ten-year fishing ban, these results establish a pre-ban trophic reference for the lower Yangtze. Future monitoring should pair abundance and diversity surveys with stable-isotope analyses to determine whether recovery brings not only more fish but also restored energy pathways and food-web function.

## Figures and Tables

**Table 1:** Species mean δ^13^C and δ^15^N values (‰) for fish collected from the Jingjiang section of the lower Yangtze River, China, during the March dry-season and August wet-season sampling campaigns in 2016. Values are reported as mean ± SD, and *n* indicates the number of individuals analysed for each species and season. Feeding-guild assignments follow regional monographs and published diet information [51]; blank cells indicate that the species was not collected in that season.

No.	Species	ID	Guild	Dry Season			Wet Season		
				δ^13^C Mean ± SD	δ^15^N Mean ± SD	*n*	δ^13^C Mean ± SD	δ^15^N Mean ± SD	*n*
1	*Coilia nasus*	Con	C	−26.56 ± 2.48	13.48 ± 2.54	8	−26.88 ± 1.13	13.94 ± 1.51	8
2	*Ctenopharyngodon idella*	Cti	H	−24.03 ± 0.05	6.44 ± 0.93	2	−25.32 ± 1.88	9.52 ± 1.08	4
3	*Cultrichthys erythropterus*	Cue	C	−21.54 ± 0.00	13.15 ± 0.00	1	−28.16 ± 1.04	11.77 ± 1.46	4
4	*Hemiculter bleekeri*	Heb	O	−28.14 ± 2.17	9.42 ± 1.70	5	−28.92 ± 2.72	9.14 ± 2.12	12
5	*Parabramis pekinensis*	Pap	H	−25.88 ± 2.36	11.82 ± 0.98	3	−25.59 ± 1.30	8.62 ± 1.34	5
6	*Pseudobrama simoni*	Pss	O	−24.37 ± 5.74	9.21 ± 2.39	8	−32.07 ± 0.44	7.65 ± 0.63	2
7	*Xenocypris argentea*	Xea	O	−25.58 ± 4.28	9.43 ± 0.90	4	−27.47 ± 6.30	7.30 ± 1.68	4
8	*Hypophthalmichthys molitrix*	Hym	H	−26.77 ± 0.80	6.30 ± 1.78	4	−30.78 ± 1.01	6.71 ± 0.94	4
9	*Aristichthys nobilis*	Arn	C	−21.19 ± 3.49	7.73 ± 1.86	4	−29.51 ± 1.45	7.28 ± 1.80	3
10	*Abbotina rivularis*	Abr	O	−24.38 ± 2.96	12.02 ± 1.21	3	−25.55 ± 3.94	11.39 ± 1.60	3
11	*Pseudorasbora parva*	Psp	O	−28.70 ± 0.80	10.96 ± 0.90	2	−26.71 ± 3.34	10.35 ± 1.80	6
12	*Saurogobio dabryi*	Sau	C	−22.56 ± 3.02	14.33 ± 0.53	3	−23.74 ± 3.95	12.36 ± 1.61	5
13	*Saurogobio gymnocheilus*	Sag	C	−22.30 ± 2.78	14.58 ± 0.93	4	−26.76 ± 0.26	14.87 ± 0.63	3
14	*Squalidus argentatus*	Sqa	O	−25.44 ± 0.72	11.31 ± 2.38	4	−26.69 ± 2.47	10.87 ± 1.69	6
15	*Acheilognathus chankaensis*	Acc	H	−24.81 ± 3.72	9.81 ± 1.16	2	−24.76 ± 0.55	10.62 ± 0.95	3
16	*Acheilognathus macropterus*	Acm	O	−24.84 ± 5.89	10.24 ± 1.51	2	−22.99 ± 0.89	9.00 ± 1.03	2
17	*Rhodeus ocellatus*	Rho	O	−25.05 ± 1.15	10.82 ± 0.37	2	−29.39 ± 2.74	10.20 ± 0.99	2
18	*Carassius auratus*	Caa	O	−24.08 ± 6.12	9.02 ± 2.27	4	−26.59 ± 2.35	11.92 ± 2.02	5
19	*Misgurnus anguillicaudatus*	Mia	O	−23.33 ± 0.82	7.26 ± 1.32	3	−26.99 ± 3.20	6.27 ± 0.97	3
20	*Pseudobagrus nitidus*	Psn	C	−25.60 ± 2.05	13.59 ± 0.89	5	−26.22 ± 0.78	12.71 ± 1.33	6
21	*Lateolabrax maculatus*	Lam	C	−23.32 ± 1.33	14.62 ± 0.93	6	−25.72 ± 1.46	12.62 ± 1.57	6
22	*Callionymus olidus*	Cao	C	−23.40 ± 2.11	13.23 ± 0.92	4	−28.99 ± 0.61	13.74 ± 0.21	2
23	*Rhinogobius giurinus*	Rhg	C	−22.36 ± 6.32	12.12 ± 3.11	5	−26.93 ± 1.19	12.14 ± 0.47	2
24	*Cynoglossus gracilis*	Cyg	O	−25.67 ± 0.38	14.67 ± 1.29	4	−25.94 ± 0.47	13.95 ± 0.92	6
25	*Hyporhamphus intermedius*	Hyi	C	−25.26 ± 3.20	13.07 ± 1.91	4			
26	*Micropercops swinhonis*	Mis	O	−30.52 ± 1.63	10.41 ± 0.85	2			
27	*Lophiogobius ocellicauda*	Loo	C	−24.42 ± 1.66	14.90 ± 0.17	2			
28	*Salanx ariakensis*	Saa	C				−20.84 ± 3.86	11.01 ± 1.54	4
29	*Salanx cuvieri*	Sac	C				−21.92 ± 2.59	10.78 ± 1.50	6
30	*Anguilla japonica*	Anj	C				−22.94 ± 3.26	13.31 ± 1.21	6
31	*Squaliobarbus curriculus*	Sqc	O				−24.39 ± 0.69	10.38 ± 0.82	2
32	*Culter alburnus*	Cua	C				−26.22 ± 2.15	12.05 ± 1.95	6
33	*Megalobrama skolkovii*	Mes	H				−26.63 ± 2.26	13.13 ± 1.03	3
34	*Pseudolaubuca engraulis*	Pse	O				−25.55 ± 0.01	9.74 ± 1.90	2
35	*Pseudolaubuca sinensis*	Pssi	O				−26.40 ± 1.61	9.78 ± 2.25	2
36	*Xenocypris davidi*	Xed	O				−27.99 ± 3.56	10.09 ± 1.65	2
37	*Sarcocheilichthys nigripinnis*	San	O				−23.36 ± 0.00	12.04 ± 0.00	1
38	*Saurogobio dumerili*	Sad	C				−23.43 ± 2.16	14.34 ± 1.11	6
39	*Cyprinus carpio*	Cyc	O				−23.52 ± 0.98	8.82 ± 1.92	3
40	*Pelteobagrus fulvidraco*	Pef	C				−23.68 ± 0.89	11.03 ± 0.98	6
41	*Pelteobagrus eupogon*	Pee	C				−24.38 ± 1.47	9.84 ± 1.21	7
42	*Silurus asotus*	Sia	C				−26.01 ± 0.00	11.34 ± 0.00	1
43	*Monopterus albus*	Moa	C				−25.86 ± 0.00	11.03 ± 0.00	1
44	*Siniperca chuatsi*	Sic	C				−26.23 ± 0.30	13.60 ± 0.43	3
45	*Siniperca kneri*	Sik	C				−27.88 ± 1.59	11.75 ± 0.87	9
46	*Siniperca scherzeri*	Sis	C				−27.04 ± 0.00	10.90 ± 0.00	1
47	*Bostrychus sinensis*	Bos	C				−28.96 ± 0.00	10.85 ± 0.00	1
48	*Eleotris oxycephala*	Elo	C				−27.12 ± 0.64	14.20 ± 1.25	2
49	*Channa argus*	Cha	C				−24.44 ± 4.33	9.23 ± 2.18	4
50	*Takifugu obscurus*	Tao	C				−23.97 ± 0.71	11.74 ± 1.24	3

Note: Two feeding-guild entries were harmonised with published diet data: *Acheilognathus macropterus* was reclassified as omnivorous and *Megalobrama skolkovii* as herbivorous. Guild abbreviations: H = herbivorous, O = omnivorous, C = carnivorous.

**Table 2:** Community-wide isotope metrics for the 24 fish species collected in both the March dry-season and August wet-season sampling campaigns in the Jingjiang section of the lower Yangtze River, China, in 2016. Metrics were calculated from unweighted species mean δ^13^C and δ^15^N coordinates. NR = δ^15^N range, CR = δ^13^C range, TA = total convex-hull area, CD = mean distance to centroid, NND = mean nearest-neighbour distance, and SDNND = standard deviation of nearest-neighbour distance.

Metric	Dry Season	Wet Season
δ^15^N range (NR)	8.37	8.60
δ^13^C range (CR)	7.51	9.08
Total convex-hull area (TA)	48.28	49.57
Mean distance to centroid (CD)	2.86	2.88
Mean nearest-neighbour distance (NND)	1.02	1.11
SD of nearest-neighbour distance (SDNND)	0.61	0.53

## Data Availability

The data supporting the findings of this study are available from the corresponding author, Hongyi Guo, hy-guo@shou.edu.cn, upon reasonable request.
